# Effect of microbubble as local drug delivery system in endodontic management - An *In-Vitro* study

**DOI:** 10.1016/j.sdentj.2024.03.010

**Published:** 2024-03-20

**Authors:** V. Shyam Ganesh, K. Vijay Venkatesh, D. Sihivahanan, Pradeep Kumar Yadalam, Deepti Shrivastava, Kumar Chandan Srivastava

**Affiliations:** a Department of Conservative Dentistry and Endodontics, SRM Kattankulathur Dental College and Hospital, SRM Institute of Science and Technology, Kattankulathur, Tamil Nadu 603203, India; b Department of Periodontics, Saveetha Dental College and Hospitals, Saveetha Institute of Medical and Technical Sciences, Saveetha University, Chennai, Tamil Nadu, India; c Department of Preventive Dentistry, College of Dentistry, Jouf University, Sakaka, Saudi Arabia; d Department of Oral & Maxillofacial Surgery & Diagnostic Sciences, College of Dentistry, Jouf University, Sakaka, Saudi Arabia; e Department of Oral Medicine and Radiology, Saveetha Dental College, Saveetha Institute of Medical and Technical Sciences, Saveetha University, Chennai 602105, India

**Keywords:** Microbubble, Triple antibiotic paste, *Enterococcus faecalis*, Intra canal medication, and Antibacterial efficacy

## Abstract

**Background and Objectives:**

Microbubbles (MBs) are gas or vapor-filled cavities inside liquids with sizes ranging from 2 to 3 µm. Recently, MBs have shown great promise in nanomedicine owing to their high encapsulation efficiency, targeted drug release, improved biocompatibility, and longer blood circulation. Furthermore, they are more suitable for focusing on particular body regions and are safer and non-invasive. MBs generators are used to create bubbles in fluid dynamics, chemistry, medicine, agriculture, and the environment. Drug delivery using MBs increases penetration without causing systemic toxicity. In this study, we examined whether the use of microbubbles as a local drug-delivery mechanism increases tubular penetration of endodontic medications and irrigant.

**Materials and Methods:**

An *Enterococcus faecalis* culture was added to 38 dentin cylinders of single-rooted teeth. Samples were divided into the experimental and control groups that received a triple antibiotic paste with and without MB infusion (n = 19 in each group), respectively. After 14 days, the number of live bacteria in the samples was determined using confocal laser scanning microscopy.

**Results:**

After 14 days of contact with the medication, the percentages of live and dead bacteria were assessed. Results show that Group 2 (Triple antibiotic infused micro bubble) showed significantly (P < 0.05) higher antibacterial efficacy than Group 1 (TAP).

**Conclusion:**

In this study, the antibacterial efficacy was significantly higher in the experimental group than in the control group. Therefore, within the limitations of the study it can be said that MB infusion is a viable technique to improve root canal disinfection. Hence, it can be considered as a novel technique for local drug delivery systems in endodontic management.

## 1 Introduction

Bacteria in the root canal system is the primary factor in developing apical periodontitis of endodontic origin (Janani,Teja,and Srivatsava, 2023). The success of treatment for endodontic infections hinges on eliminating the microorganisms responsible for the infection (Teja et al., 2021). Certain microorganisms are resistant to the cleaning and shaping processes and can continue to thrive within anatomical intricacies, which presents a challenge for clinical management (Chueh and Huang, 2006). Persistent periapical lesions often contain Enterococcus faecalis because of the bacterium's propensity to colonize in dentinal tubules at varying depths (Teja et al., 2022). Effective antimicrobial treatments are necessary for a defined period to ensure the cumulative elimination of root canal bacteria (Banchs and Trope, 2004).

The European Society of Endodontists and the American Association of Endodontists advise utilizing antibiotic pastes made of two or three different antibiotics, or calcium hydroxide (CH) paste, to eliminate intracanal microbial colonies (Ribeiro et al., 2020). Triple antibiotic paste (TAP) is traditionally prepared by diluting minocycline, metronidazole, and ciprofloxacin using propylene glycol. It has been demonstrated to be more effective than pastes that contain two antibiotics, such as metronidazole and ciprofloxacin (Devaraj,Jagannathan,and Neelakantan, 2016). Due to the possibility of discoloration of minocycline, clindamycin is frequently added to TAP formulations in place of minocycline. Despite the excellent efficacy of clindamycin-modified TAP (mTAP) against endodontic infections, concerns remain regarding the penetrability of these antibiotic pastes (Karczewski et al., 2018).

In nanomedicine, microbubbles (MBs) have shown much promise in recent years because of their high encapsulation efficiency, targeted drug release, improved biocompatibility, and longer blood circulation time (Zhou et al., 2010, Lal et al., 2021). However, devices have to be designed to fabricate MBs at chair side. Microbubble are gas- or vapor-filled cavities inside liquids with diameters ranging from 2 to 3 µm (Temesgen et al., 2017). Microbubble generators can create these bubbles, which have several uses in fluid dynamics, chemistry, medicine, agriculture, and the environment (Temesgen et al., 2017, Ezike et al., 2023). Microbubbles have been employed in drug delivery systems to increase drug penetration while avoiding systemic toxicity. Furthermore, they are more suitable for focusing on particular body regions and are safe and non-invasive (Argenziano et al., 2017). To increase their capacity for tubular penetration, endodontic irrigants, and medications may eventually incorporate microbubbles (Wang et al., 2006, Dubey et al., 2009). Therefore, this study's purpose is to use qualitative and quantitative techniques to examine the impact of MB infusion on the antibacterial efficacy of intra-canal medications.

## 2 Materials and methods

This study was conducted out at SRM kattankulathur Dental College and Hospital with approval from the university's ethical committee. (EHICS CLEARANCE NUMBER: SRMIEC-ST0123-495).

### 2.1 Synthesis and characterization of Triple antibiotic-infused MBs

The most popular method for producing microbubbles is the probe sonication technique, while there are other methods as well, including spray drying, cross-linking polymerization, emulsification, emulsion solvent evaporation, and mechanical agitation (Manjanna,Shivakumar,and Kumar, 2010).

The first step of the probe-type sonication method is adding a polymer to the drug. The polymer we used is Egg white mixed with Triple antibiotic paste, which comprises metronidazole, ciprofloxacin, and minocycline; vehicle: propylene glycol at 1 1: 1 ratio (Maghsoodi, 2009). Following this, Deionized water is added to the combination and undergoes Probe sonication using a Probe sonicator to form a primary emulsion. Once the primary emulsion is formed, Dithiothreitol is added as a surfactant to improve stability by reducing the surface tension between the bubbles. After adding a surfactant, the mixture again undergoes probe sonication to form a secondary emulsion. The secondary emulsion then undergoes centrifugation and lyophilization process where oxygen gas is added to synthesize a drug-loaded Microbubble.

### 2.2 Morphological investigation of TAP-infused MBs

**Fig. 1:**
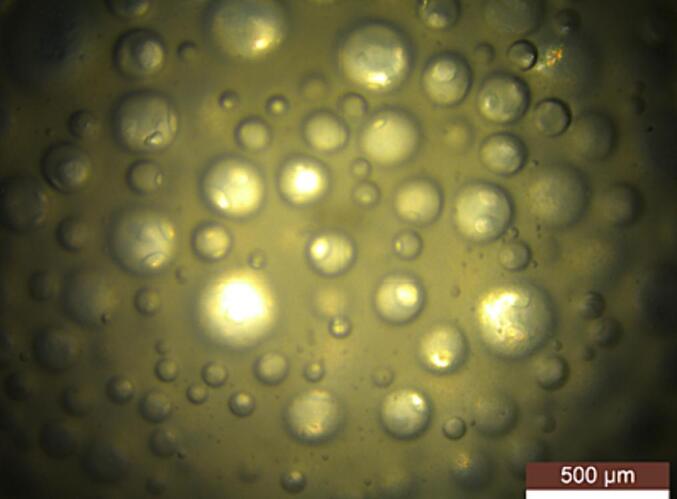
Morphological investigation of Triple antibiotic infused Microbubble done using FE- SEM.

A field emission scanning electron microscope (FE-SEM) was used to examine the size and shape of the generated TAP-infused MBs. To prepare the samples for FE-SEM analysis, a 0.5 mL drop of the sample suspension was deposited on a glass plate of fluorine-doped tin oxide and the samples were carefully dried in a desiccator for an entire night. A 4 kV FE-SEM was used to evaluate the samples following the sputtering of a thin layer of gold. (Quanta, FEG 200 High Resolution FE-SEM, FEI, Netherlands)(Maghsoodi, 2009). (Fig. 1).

### 2.3 Sample size calculation: Preparation

Considering the nature of the study, the parameters including α, sample size, and effect size were entered in the G*Power software (latest ver. 3.1.9.7; Heinrich-Heine-Universität Düsseldorf, Düsseldorf, Germany) to compute the power of the study. To compare of the means of two independent groups, a one-tailed *t* test was selected with an effect size of 0.8, α of 0.05, and equal allocation of teeth to the study groups (19 each). Considering the given parameters, the power of study was 0.78 (Kang, 2021).

### 2.4 Sample preparation

A double-sided diamond disc coupled to a low-speed handpiece under water cooling was used to remove the apical thirds of the roots of 38 maxillary central incisors to standardise 8 mm dentin cylinders. A Peeso reamer endodontic drill # 3 was used to prepare the root canals, or the internal diameter of the dentin cylinders, standardizing a diameter of 1.1 mm. The samples were autoclaved at 121 °C for 20 min.

### 2.5 Intratubular bacterial contamination

*E. faecalis* suspension was made from bacteria cultivated in brain heart infusion for 24 h using a sterile sampler microtip. It was then injected into each canal up to the orifice level. To allow the bacteria to colonize the canal wall and enter the dentinal tubules, each sample was placed in a microtube containing BHI and kept at 37 °C for 14 days in 100 % humidity. A 3 mL of BHI medium was replaced every three days. When the specimens were taken out of the inoculation tubes after 14 days, the root apices were temporarily filled with a cavity to prevent sample contamination. After contamination, the specimens were mounted in a sterile aluminum apparatus and then randomly divided into two groups.

**Group 1-**TAP; metronidazole, ciprofloxacin, and minocycline; vehicle: propylene glycol at 1: 1: 1 ratio (Sain et al., 2018).

**Group 2**- Triple antibiotic-infused Microbubble.

A syringe was used to inject the medication into the dentin cylinders until it was filled. Following the insertion of the medications, a temporary sealant was applied to the ends of the dentin cylinders. The samples were kept in Petri dishes covered with gauze dipped in distilled water and then heated to 370C in a bacteriological oven.

### 2.6 Confocal laser scanning microscopy (CLSM) analysis

**Fig. 2:**
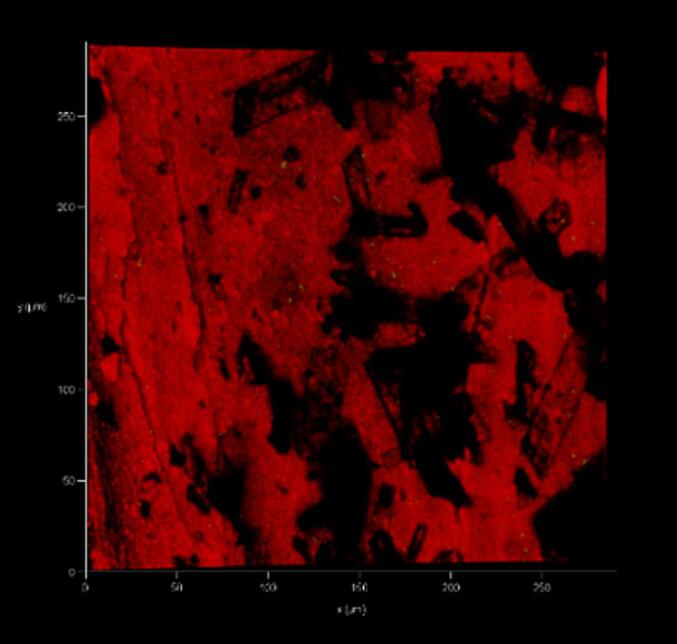
Representative confocal laser scanning microscopic images of dentinal tubules (near the root canal) of Group 1 (Triple antibiotic paste) showing both viable and dead cells. Green- viable cells; red- dead cells. (For interpretation of the references to colour in this figure legend, the reader is referred to the web version of this article.)

**Fig. 3:**
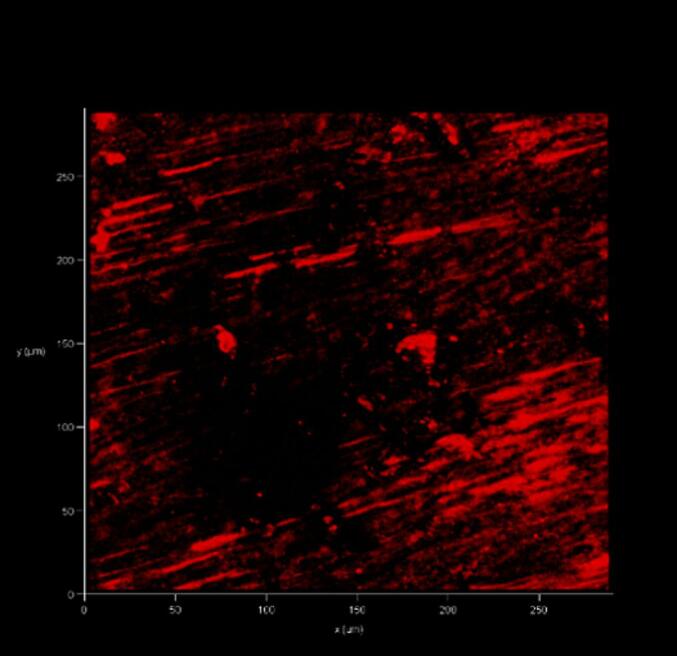
Representative confocal laser scanning microscopic images of dentinal tubules (near the root canal) of Group 2 (Triple antibiotic infused Microbubble) showing only dead cells. Green- viable cells; red- dead cells. (For interpretation of the references to colour in this figure legend, the reader is referred to the web version of this article.)

After 14 days, the temporary sealer was removed, and 5 mL of sterile distilled water was used to flush out the drug. Using a diamond disc that had been sterilized and cooled using saline solution, the specimens were longitudinally sectioned, and one of the halves was chosen randomly for analysis. Specimens were stained for 15 min using the LIVE/DEAD Back Light Bacterial Viability Kit before observation under the Zeiss LSM 510 confocal microscope for CLSM imaging (Fig. 2, Fig. 3).

### 2.7 Statistical analysis

GraphPad Prisma 8.3 Software (GraphPad Software Inc, La Jolla, CA) was used to perform the statistical analysis. The findings were determined using the percentage of viable cells from each sample. According to the Shapiro-Wilk normality test, normal distribution of the data was not present. The Kruskal-Wallis and Dunn tests were used to compare various drugs. The threshold for significance was set at P < 0.05.

## 3 Results

**Fig. 4:**
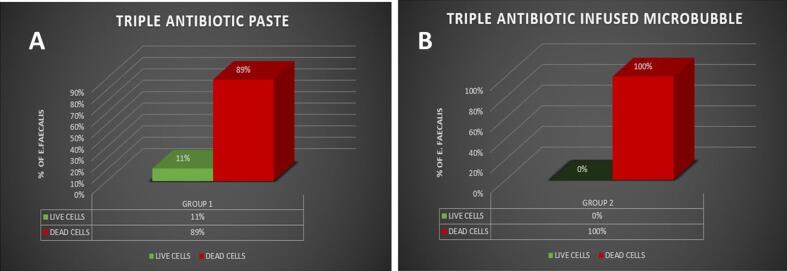
(A & B)- Graphical representation shows each group's viable bacteria percentage. Group 1 (TAP) group shows 89% of dead cells and 11% live cells, whereas, Group 2 (triple antibiotic infused micro bubble) shows 100% dead cells with no viable cells.

After 14 days of contact with the medication, the percentages of live and dead bacteria were assessed using CLSM analysis. The results showed that Group 2 (triple antibiotic infused micro bubble) had significantly higher antibacterial efficacy than Group 1 (TAP). **(**Fig. 4**A & B)** Group 1 showed that 89 % of cells were dead and 11 % were still viable, whereas, Group 2 showed that 100 % of cells were dead.

## 4 Discussion

Endodontics is one area where microbubble technologies are gaining popularity in medicine and dentistry. The utilization of extremely tiny bubbles, frequently on the micron or nanometer scale, for therapeutic or diagnostic purposes is referred to as microbubble technology. The root canal system can be filled with MBs and an appropriate irrigation fluid (Klibanov, 2008). These MBs can create mechanical forces that assist in the dissolution of biofilm, debris, and bacteria inside the intricate anatomy of the root canal when subjected to ultrasound waves. This could improve the efficiency of endodontic treatments and the success rate of root canal cleaning. Therapeutic medicines, such as antibiotics or disinfectants, can be delivered into the root canal system using MBs as carriers (Seo et al., 2010). These MBs can be filled with the required drug using ultrasound or other imaging methods and then directed to the treatment area. The MBs can release the medication once they've reached the desired size, making the treatment of infections more exact and focused. Cavitation, another name for the collapse of MBs, creates shock waves and localized pressure changes that can aid in cleaning and disinfecting the root canal system. In microspaces, MBs can intensify bubble dynamics. Microbubbles are made up of microscopic droplets that are formed in an emulsion by an oxidizer and an oxygen carrier, and have a protein/surfactant shell (Klibanov, 2008). This effect might enhance the endodontic procedure's mechanical cleaning and disinfecting procedure.

Intracanal medications are mostly utilized as an antibacterial agent to eradicate any remaining bacteria following the infected root canal's chemomechanical preparation. They are used to breakdown tissue, get rid of any bacteria that are still in the canal, create a barrier to stop microleakage, and lessen fluid seepage from the periapical area into the root canal system (Govindaraju et al., 2021).

The current study aims to increase intracanal medication’s effectiveness in intratubular disinfection of root canals by applying and testing MBs technologies for endodontic disinfection. The antibiotic must be effective in removing the endodontic biofilm while without harming the cells that will eventually form new tissue or inducing an inflammatory reaction that can impede the healing process. Given that the cytotoxicity of an antibiotic is directly related to its concentration, regenerative therapy may be adversely affected by larger dosages of the drug (Ruparel et al., 2012). According to earlier comparable *in vitro* investigations, the antibacterial effectiveness was evaluated using *Enterococcus faecalis* biofilm (Ma et al., 2011). Gram-positive facultative anaerobic bacteria, *Enterococcus faecalis*, is resistant to several medicines and possesses numerous genes linked to drug resistance. It can also endure high pH levels (Seneviratne et al., 2017). Furthermore, as a crucial component of bacterial resistance, *Enterococcus faecalis*, like the majority of bacteria connected to endodontic infections, has the ability to penetrate and colonise dentinal tubules (El-Tayeb et al., 2019). A detailed assessment of the microbial decrease within dentinal tubules was conducted using confocal laser scanning microscopy (Andrade et al. 2015; Pereira et al., 2021). With the help of confocal microscopy, it is possible to obtain sharp images with little out-of-focus interference from a narrow portion of a thick sample. With a confocal microscope and three-dimensional analysis, the contents and spatial organization of the biofilm within the dentinal tubules in various planes of the dentin mass can be seen. Confocal lasers can penetrate soft tissue up to 23 mm and tubular dentin about 15 mm, which is a significant dentin mass when one considers teeth with a single root (Pereira et al., 2021, Ma et al., 2011).

Gas-filled bubbles in the micron range formed in an emulsion are known as MBs and are frequently utilized as carriers for delivering specific drugs. These MBs originate near the liquid surface, but because of their small size, they don't burst immediately; instead, they stay in the liquid and explode there (Temesgen et al., 2017). They are also incredibly stable; they won't burst out all at once and can hold their size in liquid for months (Bhargawa et al., 2022, Klibanov, 2008). The results of this study showed that Triple antibiotic infused with MBs showed higher antibacterial efficacy than conventional TAP. The possible explanation for the improved efficacy of triple antibiotic infused MBs is due to the microbubble size, which facilitates the drug's deeper delivery into the dentinal tubules (Seo et al., 2010). The main limitation of this study is the TAP-infused MBs was in stable condition only for 23 days. Hence, future studies can focus on improving the micro bubble's stability and making it available on chair side.

## 5 Conclusion

The conclusion that MBs looks to be a potential strategy to improve root canal disinfection can be drawn from this investigation's findings and constraints. As a result, MBs infusion is regarded as a novel method of local drug administration in endodontic treatment.

## Ethical statement

The study was approved by the ethical committee of SRM University, India and was conducted at SRM kattankulathur Dental College and Hospital. (EHICS CLEARANCE NUMBER: SRMIEC-ST0123-495).

## CRediT authorship contribution statement

**V. Shyam Ganesh:** Funding acquisition, Writing – review & editing, Writing – original draft, Visualization, Validation, Data curation, Formal analysis, Investigation, Methodology, Conceptualization. **K. Vijay Venkatesh:** Project administration, Funding acquisition, Writing – review & editing, Writing – original draft, Validation, Investigation, Conceptualization, Methodology. **D. Sihivahanan:** Funding acquisition, Writing – review & editing, Validation, Conceptualization, Methodology. **Pradeep Kumar Yadalam:** Visualization, Writing – review & editing, Validation, Investigation. **Deepti Shrivastava:** Writing – review & editing, Visualization, Validation, Data curation, Methodology. **Kumar Chandan Srivastava:** Project administration, Writing – original draft, Data curation, Formal analysis, Conceptualization, Methodology, Validation, Writing – review & editing.

## Declaration of competing interest

The authors declare that they have no known competing financial interests or personal relationships that could have appeared to influence the work reported in this paper.
